# Acknowledging the peer reviewers of *Journal of Medical Radiation Sciences*, October 2021 – September 2022

**DOI:** 10.1002/jmrs.624

**Published:** 2022-11-07

The editorial review board, the Australian Society of Medical Imaging and Radiation Therapy (ASMIRT) and the New Zealand Institute of Medical Radiation Technology (NZIMRT) acknowledge the following peer reviewers for sharing their valuable time and knowledge. Thank you for your rigorous reviews.

## Outstanding Reviewers (Contributed to Five Reviews)

James Stanley

Kamarul Abdullah

Sibusiso Mdletshe

## The Following Reviewers Contributed to Four Reviews

Andrew Kilgour

Maram Alakhras

## The Following Reviewers Contributed to Three Reviews

Catriona Hargrave

Dean Paterson

Eric Pei Ping Pang

Edel Doyle

Gay Dungey

Georgia Halkett

Hesta Friedrich‐Nel

John Robinson

Kelly Lloyd

Michael Neep

Marilyn Baird

Nigel Anderson

Peter O'Reilly

Sally Ayesa

Vicki Braithwaite

## The Following Reviewers Contributed to Two Reviews

Theophilus Akudjedu

Emel Allan

Farhannah Aly

Vikneswary Batumalai

Crispen Chamunyonga

Jillian Clarke

Jenna Dean

Christopher Edwards

Rhys Fitzgerald

Therese Gunn

Peter Hogg

Melissa James

Yobelli Jimenez

Scott Jones

Stephen Knight

Heather Lawrence

Chandra Makanjee

Tracey Pieterse

Ann Poulos

Christopher Rumley

Daniel Sapkaroski

Meegan Shepherd

Clare Singh

Tony Smith

Tom Steffens

Zhonghua Sun

Rhonda‐Joy Sweeney

Caitlin Tu

Xiaoming Zheng

## The Following Reviewers Contributed to One Review

Alison Brown

Amy Brown

Allison Dry

Aileen Duffton

Daniel Al Mouiee

Fahim Alam

Christine Albantow

Essam Alkhybari

Wijdan Alomaim

Jennife Alphonse

Kholoud Alzyoud

Saba Ansari

Simon Ashworth

John Atyeo

John Baines

James Balter

Jeffrey Barber

Murat Baykara

Sarah Bergamin

Pauline Boland

Jerome Breitenberger

Mathias Bressel

Elizabeth Brown

Michael A. Bruno

Glenn Cahoon

Raphael Chee

Phillip Chlap

Thomas Cochrane

Scott Crowe

Geoffrey Currie

Gary Denham

Karen Dobeli

Laia Domingo

Kathleene Dower

Gay Dungey

Ernest Ekpo

Doaa Elwadia

Brendan Erskine

Anthony Espinoza

Giovanna Ferraioli

Karen Finlay

Ziba Gandomkar

Stuart Greenham

Christopher Hayre

Lynne Hazell

Johnathan Hewis

Bronwyn Hilder

Jonathan Hindmarsh

Sahand Hooshmand

Lin‐Fen Hsieh

Julie Hughes

Hing Hung

Manish Jain

Hazel Jenkins

Michael Jenkins

Rebecca Jude

Paul Kane

Maeve Kearney

Alannah Kejda

Peter Kench

Adam Kesner

Laurence Kim

Elin Kjelle,

Shivani Kumar

Ginna Lambert

Drew Latty

Wencke Lehnert

Shantel Lewis

Ee Shern Liang

Jens Loberg

Paul Lockwood

Elsebeth Lynge

Sharon Maresse

Kristie Matthews

Lachlan McDowell

Sonyia McFadden

Matthew McGrail

John McInerney

Stuart More

Andrew Murphy

Kathleen Naidoo

Glen Newman

Curtise K.C. Ng

Sweet Ping Ng

Henrik Nilsson

Hyunsoo No

Don Nocum

Katrina O'Keefe

Marcus Oliveira

Rachael O'Rourke

Vanessa Panettieri

Premal Patel

Alison Pearce

Klas Pekkari

Benjamin Peterson

Natalie Pollard

Owen Raffel

Clare Rainey

Gita Ralleigh

Majella Russo

Michael Savage

Laurel Schmidt

Michal Schneider

Anna Seeley

Madeleine Shanahan

Mihir Shanker

Trishna Shimpi

Michala Short

Lucy Sim

Lawrie Skinner

Beverly Snaith

Sergio Solís Barquero

Kelly Spuur

Deborah Starkey

Adam Steward

Kate Stewart

M'oayyad Suleiman

Kristie Sweeney

Hitoshi Takeuchi

Jo Thorogood

Sylvia Van Dyk

Stefaan Vandenberghe

Luke Webb

Nick Woznitza

Akira Yamada

Michael Ying

Adrienne Young

## Peer Reviewer Resources

Are you new to conducting and writing peer reviews? Check out these free peer reviewer guidelines and self‐directed modules and assess the quality of your peer review.

- Wiley Journal Reviewers

https://authorservices.wiley.com/Reviewers/journal-reviewers/index.html

- Wiley Researcher Academy: Becoming a Peer Reviewer

https://upskillstudio.researcher.life/p/wiley-researcher-academy-becoming-a-peer-reviewer

- Web of Science Academy

https://clarivate.com/webofsciencegroup/solutions/web-of-science-academy/

## Peer Reviewer Recognition

JMRS reviewers can opt in to have their peer review contribution automatically added on Web of Science Reviewer Recognition Services (formerly Publons). The authors remain confidential when the peer review contribution is recorded.

For more information please go to, https://authorservices.wiley.com/Reviewers/journal-reviewers/recognition-for-reviewers/publons.html

